# Supplementary material for: A Systematic Review and Meta-Analysis of the Impact of Mindfulness Based Interventions on Heart Rate Variability and Inflammatory Markers
Source: J Clin Med. 2019 Oct 7;8(10):1638. doi: 10.3390/jcm8101638 (PMC6833066; doi:10.3390/jcm8101638)
Supplement: Supplementary file 1 [file jcm-08-01638-s001.pdf]

Documentation of search strategies
University Library search consultation group

Date: November 2017

Topic/research question: The effects of Mindfulness Based Stress Reduction and Mindfulness based Cognitive Therapy on biological and physiological markers

Name of researcher(s): Anna Sidorchuk & Maria Niemi, Department of Public Health Sciences, Karolinska Institutet

Librarian(s): Carl Gornitzki & Klas Moberg, University Library

Databases:

1. Medline (Ovid)
2. PsycInfo (Ovid)
3. Web of Science
4. Cinahl (Ebsco)
5. Embase (embase.com)
6. PubMed (NOT medline[sb])

Grey Literature:

1. ProQuest Dissertations and Theses = 143
2. ClinicalTrials.gov = 974

Total number of hits:

- Before deduplication: 3,501 (not including grey literature)
- After deduplication: 2,324 (not including grey literature)

Comments:

Karolinska Institutet

1. Medline (Ovid)

Date of Search: 2017-11-09	Field tags:<br>• .ti,ab,kf = title, abstract & keyword<br>• exp/ = MeSH, exploded<br>• adj3 = within three words

MINDFULNESS

1. Mindfulness/
2. Meditation/
3. Mind-Body Therapies/
4. (mindfulness or meditat* or mbsr or mbct).ti,ab,kf.
5. or/1-4

BIOLOGICAL AND PHYSIOLOGICAL MARKERS

6. exp Cytokines/
7. exp Biomarkers/
8. exp Neuropeptides/
9. exp C-Reactive Protein/
10. exp Heart Rate/
11. exp Autonomic Nervous System/
12. exp Vagus Nerve/
13. exp Insulin-Like Growth Factor I/
14. exp Stress, Physiological/
15. (cytokine* or interleukin* or biological marker* or biomarker* or neuropeptide* or c-reactive protein* or crp or variability or autonom* nervous system or ans or vagal activity or vagus activity or insulin-growth factor* or insulin-like growth factor* or physiological stress or physiological activit*).ti,ab,kf.
16. (((heart or puls*) adj3 (beat* or rate*)) or (heartrate* or heartbeat*)).ti,ab,kf.
17. or/6-16

18. 5 and 17
19. remove duplicates from 18

Karolinska Institutet

2. PsycInfo (Ovid)

Date of Search: 2017-11-09	
1. Mindfulness/<br>2. Meditation/<br>3. Mind Body Therapy/<br>4. (mindfulness or meditat* or mbsr or mbct).ti,ab,id.<br>5. or/1-4<br><br>6. exp Cytokines/<br>7. Biological Markers/<br>8. exp Neuropeptides/<br>9. Heart Rate/<br>10. exp Autonomic Nervous System/<br>11. Vagus Nerve/<br>12. Insulin-like Growth Factor/<br>13. Physiological Stress/<br>14. (cytokine* or interleukin* or biological marker* or biomarker* or neuropeptide* or c-reactive protein* or crp or variability or autonom* nervous system or ans or vagal activity or vagus activity or insulin-growth factor* or insulin-like growth factor* or physiological stress or physiological activit*).ti,ab,id.<br>15. (((heart or puls*) adj3 (beat* or rate*)) or (heartrate* or heartbeat*)).ti,ab,id.<br>16. or/6-15<br><br>17. 5 and 16	

3. Web of Science

Date of Search: 2017-11-09	Field tags:<br><br>• TS = Topic: title, abstract, keywords

TS=(mindfulness OR meditat* OR mbsr OR mbct)

AND

TS=(cytokine* OR interleukin* OR "biological marker*" OR biomarker* OR neuropeptide* OR c-reactive protein* OR crp OR variability OR "autonom* nervous system" OR ans OR "vagal activity" OR "vagus activity" OR "insulin-growth factor*" OR "insulin-like growth factor*" OR "physiological stress" OR "physiological activit*") OR TS=(((heart OR puls*) NEAR/3 (beat* OR rate*)) OR (heartrate* OR heartbeat*))

4. Cinahl (Ebsco)

Date of Search: 2017-11-09	Field tags:<br>• MH = Cinahl Headings
S17	S5 AND S16
S16	S6 OR S7 OR S8 OR S9 OR S10 OR S11 OR S12 OR S13 OR S14 OR S15
S15	TI ((((heart OR puls*) N3 (beat* OR rate*)) OR (heartrate* OR heartbeat*))) OR AB ((((heart OR puls*) N3 (beat* OR rate*)) OR (heartrate* OR heartbeat*)))
S14	TI ((cytokine* OR interleukin* OR "biological marker*" OR biomarker* OR neuropeptide* OR c-reactive protein* OR crp OR variability OR "autonom* nervous system" OR ans OR "vagal activity" OR "vagus activity" OR "insulin-growth factor*" OR "insulin-like growth factor*" OR "physiological stress" OR "physiological activit*")) OR AB ((cytokine* OR interleukin* OR "biological marker*" OR biomarker* OR neuropeptide* OR c-reactive protein* OR crp OR variability OR "autonom* nervous system" OR ans OR "vagal activity" OR "vagus activity" OR "insulin-growth factor*" OR "insulin-like growth factor*" OR "physiological stress" OR "physiological activit*"))
S13	(MH "Stress, Physiological")
S12	(MH "Vagus Nerve+")
S11	(MH "Autonomic Nervous System+")
S10	(MH "Heart Rate+")
S9	(MH "C-Reactive Protein")

S8	(MH "Neuropeptides+")
S7	(MH "Biological Markers+")
S6	(MH "Cytokines+")
S5	S1 OR S2 OR S3 OR S4
S4	TI ((mindfulness or meditat* or mbsr or mbct)) OR AB ((mindfulness or meditat* or mbsr or mbct))
S3	(MH "Mind Body Techniques")
S2	(MH "Meditation")
S1	(MH "Mindfulness")

Karolinska Institutet

5. Embase (embase.com)

Date of Search: 2017-11-09	Field tags:<br>• :ab,ti = title & abstract<br>• /exp = Emtree, exploded<br>• /de = Emtree, non-exploded

No.	Query	Results
#17	#4 AND #16	990
#16	#5 OR #6 OR #7 OR #8 OR #9 OR #10 OR #11 OR #12 OR #13 OR #14 OR #15	2,333,547
#15	((heart OR puls*) NEAR/3 (beat* OR rate*)):ab,ti OR heartrate*:ab,ti OR heartbeat*:ab,ti	199,113
#14	cytokine*:ab,ti OR interleukin*:ab,ti OR 'biological marker*':ab,ti OR biomarker*:ab,ti OR neuropeptide*:ab,ti OR 'c reactive':ab,ti ANDprotein*:ab,ti OR crp:ab,ti OR variability:ab,ti OR 'autonom* nervous system':ab,ti OR ans:ab,ti OR 'vagal activity':ab,ti OR 'vagus activity':ab,ti OR 'insulin-growth factor*':ab,ti OR 'insulin-like growth factor*':ab,ti OR 'physiological stress':ab,ti OR 'physiological activit*':ab,ti	608,127
#13	'physiological stress'/de	12,232
#12	'somatomedin c'/de	43,461
#11	'vagus nerve'/de	20,174
#10	'autonomic nervous system'/exp	240,375
#9	'heart rate'/exp	204,453
#8	'c reactive protein'/de	110,290

Karolinska Institutet

#7	'neuropeptide'/exp	171,297
#6	'biological marker'/de	171,785
#5	'cytokine'/exp	1,146,517
#4	#1 OR #2 OR #3	10,057
#3	mindfulness:ab,ti OR meditat*:ab,ti OR mbsr:ab,ti OR mbct:ab,ti	8,107
#2	'meditation'/de	4,823
	'mindfulness'/de	1,945

5. PubMed (NOT medline[sb])

Date of Search: 2017-11-09	
(((((((heart[Title/Abstract] OR puls*[Title/Abstract])) AND (beat*[Title/Abstract] OR rate*[Title/Abstract]))) OR ((cytokine*[Title/Abstract] OR interleukin*[Title/Abstract] OR "biological marker*"[Title/Abstract] OR biomarker*[Title/Abstract] OR neuropeptide*[Title/Abstract] OR c-reactive protein*[Title/Abstract] OR crp[Title/Abstract] OR variability[Title/Abstract] OR "autonomic nervous system"[Title/Abstract] OR ans[Title/Abstract] OR "vagal activity"[Title/Abstract] OR "vagus activity"[Title/Abstract] OR "insulin-growth factors"[Title/Abstract] OR "insulin-like growth factors"[Title/Abstract] OR "insulin-growth factor"[Title/Abstract] OR "insulin-like growth factor"[Title/Abstract] OR "physiological stress"[Title/Abstract] OR "physiological activit*"[Title/Abstract] OR heartrate*[Title/Abstract] OR heartbeat*[Title/Abstract])))) <br>AND <br>((mindfulness[Title/Abstract] OR meditat*[Title/Abstract] OR mbsr[Title/Abstract] OR mbct[Title/Abstract]))) <br>NOT <br>medline[sb]	
